# Development of a novel and economical agar-based non-adherent three-dimensional culture method for enrichment of cancer stem-like cells

**DOI:** 10.1186/s13287-018-0987-x

**Published:** 2018-09-26

**Authors:** Weijie Gao, Dinglan Wu, Yuliang Wang, Zhu Wang, Chang Zou, Yong Dai, Chi-Fai Ng, Jeremy Yuen-Chun Teoh, Franky Leung Chan

**Affiliations:** 1 School of Biomedical Sciences, Faculty of Medicine, The Chinese University of Hong Kong, Shatin, Hong Kong China; 2 Shenzhen Key Laboratory of Viral Oncology, The Clinical Innovation & Research Center, Shenzhen Hospital, Southern Medical University, Shenzhen, 518110 China; 3 Clinical Medical Research Center, The Second Clinical Medical School of Jinan University, Shenzhen People’s Hospital, Shenzhen, 518000 China; 4 Department of Surgery, Faculty of Medicine, The Chinese University of Hong Kong, Hong Kong, China

**Keywords:** 3D culture, Agar gel, Anchorage-independent growth, Cancer stem-like cells, Prostatospheres, Tumor spheres

## Abstract

**Background:**

Non-adherent or ultra-low attachment three-dimensional (3D) culture, also called sphere formation assay, has been widely used to assess the malignant phenotype and stemness potential of transformed or cancer cells. This method is also popularly used to isolate the cancer stem-like cells (CSCs) or tumor-initiating cells based on their unique anchorage-independent growth or anoikis-resistant capacity. Different non-adhesive coating agents, such as poly-2-hydroxyethyl methacrylate (poly-HEMA) and synthetic hydrogels, have been used in this non-adherent 3D culture. However, preparation of non-adherent culture-ware is labor-intensive and technically demanding, and also costs of commercial non-adherent culture-ware prepared with various coating agents are relatively expensive and the culture-ware cannot be used repeatedly.

**Methods:**

In this study, we developed a non-adherent 3D culture method based on agar coating for growing tumor spheres derived from various cancer cell lines and primary prostate cancer tissues under a non-adherent and serum-free condition. The tumor spheres generated by this 3D culture method were analyzed on their expression profiles of CSC-associated markers by reverse transcription quantitative polymerase chain reaction, presence and relative proportion of CSCs by fluorescence-activated cell sorting (CD133^+^/CD44^+^ cell sorting) and also a CSC-visualizing reporter system responsive to OCT4 and SOX2 (SORE6), and *in vivo* tumorigenicity. The repeated use of agar-coated plates for serial passages of tumor spheres was also evaluated.

**Results:**

Our results validated that the multicellular tumor spheres generated by this culture method were enriched of CSCs, as evidenced by their enhanced expression profiles of CSC markers, presence of CD133^+^/CD44^+^ or SORE6^+^ cells, enhanced self-renewal capacity, and *in vivo* tumorigenicity, indicating its usefulness in isolation and enrichment of CSCs. The agar-coated plates could be used multiple times in serial passages of tumor spheres.

**Conclusions:**

The described agar-based 3D culture method offers several advantages as compared with other methods in isolation of CSCs, including its simplicity and low-cost and repeated use of agar-coated plates for continuous passages of CSC-enriched spheres.

**Electronic supplementary material:**

The online version of this article (10.1186/s13287-018-0987-x) contains supplementary material, which is available to authorized users.

## Background

It is well recognized that cancers consist of heterogeneous subpopulations of cells, which display various genotypes and phenotypes as reflected in their diverse clinical behaviors and potential in tumor development, metastasis, relapse, and resistance to therapy. There is a small subpopulation of cancer cells present in cancers or solid tumors referred to as cancer stem cells (SCs) or cancer stem-like cells (CSCs) (also called tumor-initiating or cancer progenitor cells), that is based on their certain characteristic growth features commonly sharing with the normal tissue SCs or progenitor cells, including self-renewal, resistance to apoptosis and anticancer drugs, differentiation and high tumor regeneration capacity when grown *in vivo* [1]. Although CSCs are rare within the tumor mass [2], they can be identified and isolated from many solid tumors and their derived cancer cell lines, including brain, breast, colon, lung, pancreas, and prostate. Accumulating evidence indicates that these CSCs contribute significantly to cancer initiation and recurrence, resistance to most therapies, and metastasis in advanced cancer development [3–6]. Targeting CSCs is becoming an attractive therapeutic strategy for treatment of advanced therapy-resistant cancers. Effective and reliable methods for CSC isolation and enrichment are crucial for their study.

Hitherto CSCs can be identified and isolated by several methodologies based on their unique growth features and stemness phenotypes, including (1) sphere formation assay or non-adherent three-dimensional (3D) culture based on the self-renewal and anchorage-independent growth potential or anoikis resistance, (2) flow cytometry–based fluorescence-activated cell sorting (FACS) and magnetic bead-based magnetic-activated cell sorting (MACS) methods by the unique expression of certain cell surface markers (for example, CD44, CD133, and α2β1 integrin), (3) reporter systems driven by SC-controlling core transcription factors (*OCT4*, *SOX2*, and *NANOG*), (4) side population (SP) cell sorting by the high drug efflux capacity, and (5) Aldefluor assay by the increased aldehyde dehydrogenase isoform 1 (ALDH1) activity [7, 8].

Among these methods, sphere formation assay or non-adherent 3D culture, which is evolved from the original non-adherent neurosphere assay for culturing free-floating neural SCs in serum-free (SF) conditions [9], is the most widely used method to isolate and enrich CSCs and also to assay stemness potential. In this method, suspended single cells are cultured at low cell density in a non-adherent or ultra-low attachment suspension culture condition or 3D cultures with different natural or synthetic supporting matrices or scaffolds (for example, Matrigel, collagen, and synthetic hydrogels) in SF defined medium and the survival cells grown as spherical aggregates or spheres are considered to be enriched of CSCs and single cell–derived [10, 11]. In scaffold-supported 3D cultures, interaction of CSCs with the natural extracellular matrices (ECMs) can maintain the stemness of CSCs and their survival [12, 13] but also may activate certain signaling pathways leading to differentiation of CSCs [14, 15]. Thus, CSC populations as acquired by ECM-based 3D cultures are heterogeneous as compared with non-adherent 3D culture and may affect their subsequent analyses. One main limitation of this method is that the dormant CSCs may not divide to form multicellular spheres [16].

CSCs can be identified on the basis of their specific expressed cell surface markers that are common to SCs or normal tissue SCs [8, 17]. Different CSC or SC-associated cell surface markers are being used to detect and isolate CSCs by the flow cytometry–based FACS and MACS methods using specific antibodies [18]. For example, some membrane markers, including CD44, CD133, CD24, and α2β1 integrin, are used successfully by these methods to isolate prostate cancer stem-like cells (PCSCs) or tumor progenitor cells from cell lines and primary tumors [19–21]. However, so far, there are still no universal CSC-specific markers identified for most cancer types. Based on the conserved feature of high drug efflux capacity and expression of efflux membrane transporters, particularly ABCG2 in SCs or SPs derived from various tissue origins [22], sorting of SP cells by exclusion of DNA dye Hoechst 33342 is another flow cytometry–based method used for isolation of CSCs with high tumorigenicity and the isolated SP cells are validated as a CD44^+^/CD133^+^ population [23–25]. Increased activity of ALDH1 is also a specific feature of SCs and CSCs [26, 27]. Based on this, a flow cytometry–based method (Aldefluor assay) is developed to isolate CSCs from different cancer tissues [28–30]. However, it is under query that high ALDH1 activity may not be a specific marker for PCSCs but only the highly tumorigenic prostate cancer cells [31]. However, all these flow cytometry–based methods have their own advantages and disadvantages. The cell injury caused by cell sorting, high costs of equipment, and specific antibodies are the concerns or limitations of their applications. Combined applications of these methods are commonly used to identify and isolate the CSCs from various sources for subsequent characterization, such as expression patterns of stemness biomarkers, sphere formation capacity, and tumor formation capacity in host mice. In this study, we aim to develop a low-cost agar-based non-adherent 3D culture method for the improved isolation and enrichment of CSCs and also to evaluate the repeated use of agar-coated plates in continuous suspension culture of CSCs.

## Methods

### Cell lines and monolayer cultures

A panel of cancer cell lines, including prostate cancer (LNCaP, VCaP, DU145, and 22Rv1), colorectal carcinoma (HCT116), and hepatoma (HepG2), were used in this study. All cell lines were obtained from the American Type Culture Collection (Manassas, VA, USA). For conventional two-dimensional (2D) monolayer cultures, LNCaP and 22Rv1 cells were grown in RPMI-1640; DU145 and HepG2 cells in MEM; and HCT116 cells in McCoy’s 5A, and all growth media was supplemented with 10% fetal bovine serum (FBS) and 1% penicillin-streptomycin mixture (Gibco, Thermo Fisher Scientific, Waltham, MA, USA). In addition, primary cultures of surgical prostate cancer tissues were maintained in RPMI-1640 medium supplemented with Rho kinase (ROCK) inhibitor Y-27632 under hypoxia conditions [32]. CWR22 xenograft of primary prostate cancer was serially propagated in male severe combined immunodeficiency (SCID) mice as described previously [33].

### Preparation of agar-coated plates and dishes

**Fig. 1:**
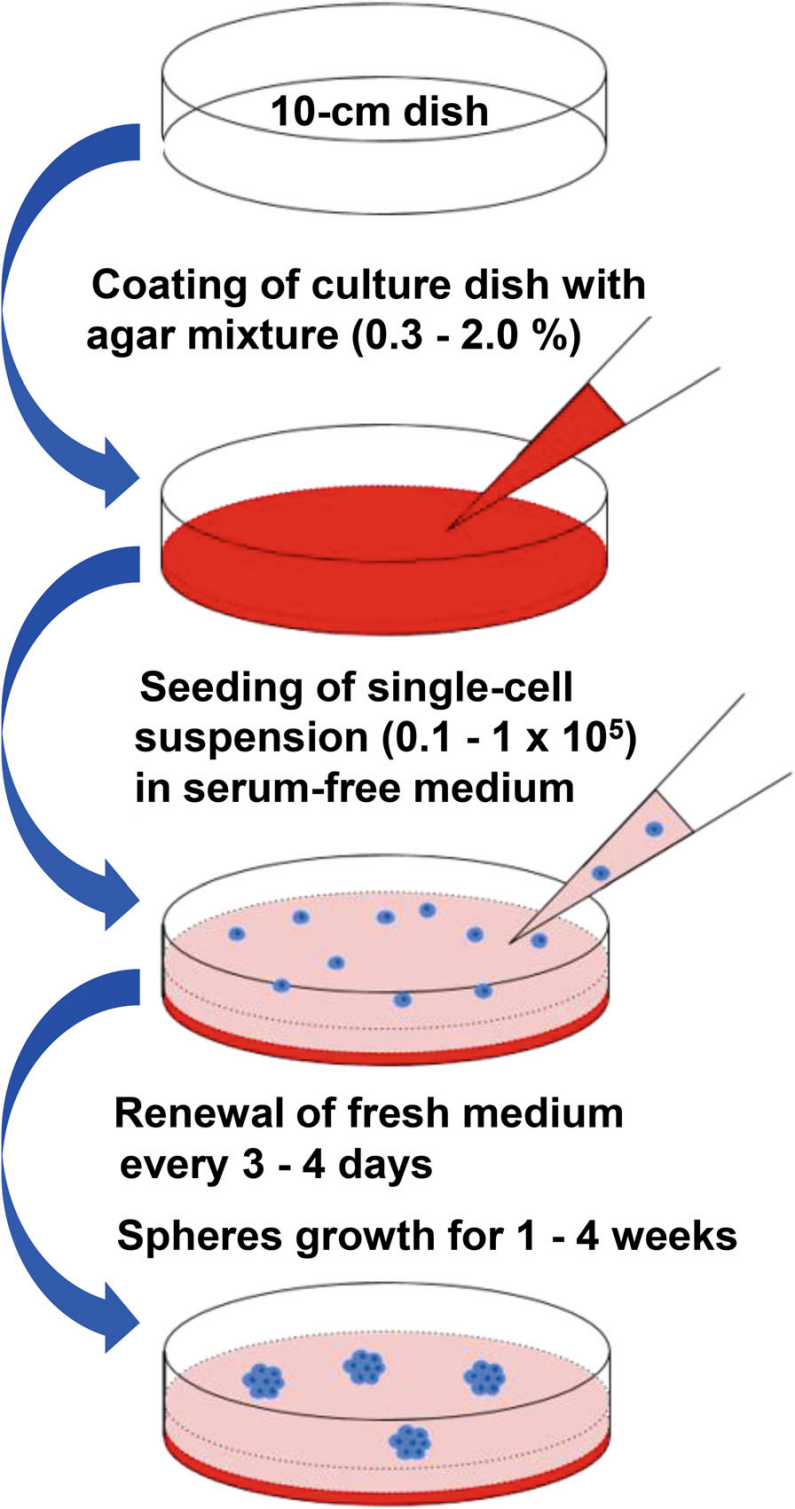
Scheme of preparation of agar-coated dishes and procedures for non-adherent three-dimensional (3D) culture

Difco™ Noble agar (BD Biosciences, San Jose, CA, USA) was dissolved in distilled water at 0.6–4.0% concentrations, and solutions were kept in a water bath at 45 °C after autoclave. The agar solutions and 2 × Dulbecco’s modified Eagle’s medium/Nutrient Mixture F-12 (DMEM/F12) medium, pre-warmed in a water bath to 45 °C, were mixed at equal volumes. The DMEM/F12-agar mixture solution was poured immediately onto culture plates or dishes with gentle swirl for even coating of culture-ware with agar solution. The agar-coated plates and dishes were placed in a culture cabinet for 15–30 min until complete solidification of agar. The final concentrations of coated agar layer were 0.3–2.0%. For prepared agar-coated plates and dishes that were not used immediately, a small volume (3–5 mL/per 10-cm dish) of SF medium—DMEM/F12 medium (GlutaMAX™; Gibco) supplemented with 20 ng/mL human epidermal growth factor (Gold Biotechnology, St. Louis, MO, USA), 20 ng/mL human basic fibroblast growth factor (Gold Biotechnology), 4 μg/mL insulin (Sigma-Aldrich, St. Louis, MO, USA), 1 × B-27 supplement (Gibco), 1% KnockOut serum replacement (Gibco) and 1% penicillin-streptomycin—was added to dishes in order to prevent the agar gel from dehydration and was sealed and kept at 4°C for 1 week or longer for subsequent uses. The coating procedure is depicted in Fig. 1.

### 3D cultures

(a) *Agar-based non-adherent 3D culture*. Single-cell suspensions were prepared by treating monolayer-cultured cancer cells or minced prostate cancer tissues with trypsin replacement reagent (TrypLE; Gibco) for 5 min with gentle shaking and rinsed with phosphate-buffered saline (PBS) to remove FBS. Cells suspended in SF medium (at density of 1 × 10^4^–1 × 10^5^ per 6–10 mL medium for 10-cm plates) were plated on agar-coated dishes or plates and cultured for 1–3 weeks for formation of spheres, and fresh medium was renewed every 3–4 days. The culture procedure is depicted in Fig. 1. Primary spheres were collected by gravity (for 3–5 min) or using 80-μm filters, dissociated into single cells by TrypLE reagent for 5 min with gentle rocking, rinsed with PBS, re-suspended in SF medium, and plated onto agar-coated dishes for continued passages. The used agar-coated dishes were washed one or two times with PBS to completely remove any remaining residual cells and ready for reuse for continued culture of the same tumor spheres. (b) *Matrigel-based 3D culture*. Overlay Matrigel-based 3D culture was performed on 48-well plates pre-coated with growth factor reduced (GFR)-Matrigel (Corning^®^, Corning, NY, USA) in accordance with a procedure described previously [34]. In brief, single-cell suspensions in SF medium were pre-cooled on ice and mixed with GFR-Matrigel (1:3 volume ratio). Cells were seeded onto Matrigel-coated wells (0.5–1.5 × 10^3^ cells per well) and incubated at 37 °C for 15 min for Matrigel solidification. Cells were 3D-cultured for 1–3 weeks and fresh medium was renewed every 4 days. Spheres formed with sizes of at least 50 μm were enumerated under microscope and collected for further analyses. The sphere formation capacity was determined by number of spheres formed per 1000 cells seeded. (c) *3D culture in ultra-low attachment dishes*. Single-cell suspensions of cancer cells were grown in commercially available ultra-low attachment dishes (Corning^®^) in accordance with the instructions of the manufacturer.

### Flow cytometry

For FACS, single-cell suspensions of 2D cultured adherent cells or 3D cultured spheres in PBS with 0.5% bovine serum albumin were incubated with fluorochrome-conjugated antibodies CD133-allophycocyanin (APC) and CD44-FITC (1:11 dilution; Miltenyi Biotec, Bergisch Gladbach, Germany) or isotype control antibodies for 10 min at 2–8 °C. The labeled cells were washed, re-suspended in PBS, and analyzed on a flow cytometer (BD LSRFortessa Cell Analyzer).

### RT-qPCR analysis

Total RNA was extracted from 2D cultured cells and 3D cultured spheres using TRIzol reagent (Molecular Research Center) in accordance with the instructions of the manufacturer, followed by reverse transcription using PrimeScript reverse transcriptase (TaKaRa Bio Inc., Kusatsu, Japan). Real-time polymerase chain reaction (PCR) was performed by using a SYBR green fluorescence-based method (SYBR Premix Ex Taq; TaKaRa Bio Inc.) as described previously [35] and in a real-time PCR system (StepOne; Applied Biosystems, Foster City, CA, USA). The sequences of primers used are listed in the Table 1.

**Table 1:** Nucleotide sequences of primers used for quantitative polymerase chain reaction analysis

Gene name	Alias	Forward (5′-3′)	Reverse (5′-3′)
*CD24*	CD24	CTCCTACCCACGCAGATTTATTC	TGGTGGCATTAGTTGGATTTGG
*CD44*	CD44	CAGCACCATTTCAACCACAC	GTTGCCAAACCACTGTTCCT
*PROM1*	CD133	AAACAGTTTGCCCCCAGGAA	ACAATCCATTCCCTGTGCGT
*KRT5*	CK5	AGGAGTTGGACCAGTCAACAT	TGGAGTAGTAGCTTCCACTGC
*KRT14*	CK14	TGAGCCGCATTCTGAACGAG	GATGACTGCGATCCAGAGGA
*NANOG*	NANOG	TTTGTGGGCCTGAAGAAAACT	AGGGCTGTCCTGAATAAGCAG
*BMI1*	BMI1	GCTGCCAATGGCTCTAATGAA	TGCTGGGCATCGTAAGTATCTT
*POU5F1*	OCT4	GACAACAATGAAAATCTTCAGGAGA	CTGGCGCCGGTTACAGAACCA
*SOX2*	SOX2	GCCGAGTGGAAACTTTTGTCG	GGCAGCGTGTACTTATCCTTCT
*CTNNB1*	β-catenin	TTGTGCGGCGCCATTTTAAG	TCCTCAGACCTTCCTCCGTC
*AR*	AR	CGGAAGCTGAAGAAACTTGG	ATGGCTTCCAGGACATTCAG
*KLK3*	PSA	TTGTCTTCCTCACCCTGTCC	TCACGCTTTTGTTCCTGATG
*KLK2*	Kallikrein 2	CTGCCCATTGCCTAAAGAAG	GCTCACACACTGAAGACTCCTG
*ACTB*	β-actin	ATGGATGATGATATCGCCGCG	CTCCATGTCGTCCCAGTTGGT

### *In vivo* tumorigenicity assay

Single-cell suspensions were prepared from 2D cultured prostate cancer cells and 3D cultured prostatospheres, mixed with Matrigel (1 × 10^3^, 10^4^, or 10^5^ cells per 100 μL mixed 1:1 Matrigel), and injected subcutaneously into the flanks of intact male SCID mice and allowed to grow for 6 weeks. Tumor growth and sizes were monitored weekly and measured as described previously [35].

### Statistical analysis

All results were expressed as mean ± standard deviation. Statistical analyses of data were performed by using two-tail Student’s *t* test, and differences were considered significant where *P* value was less than 0.05.

## Results

### Establishment of an agar-based non-adherent 3D culture of cancer cells

**Fig. 2:**
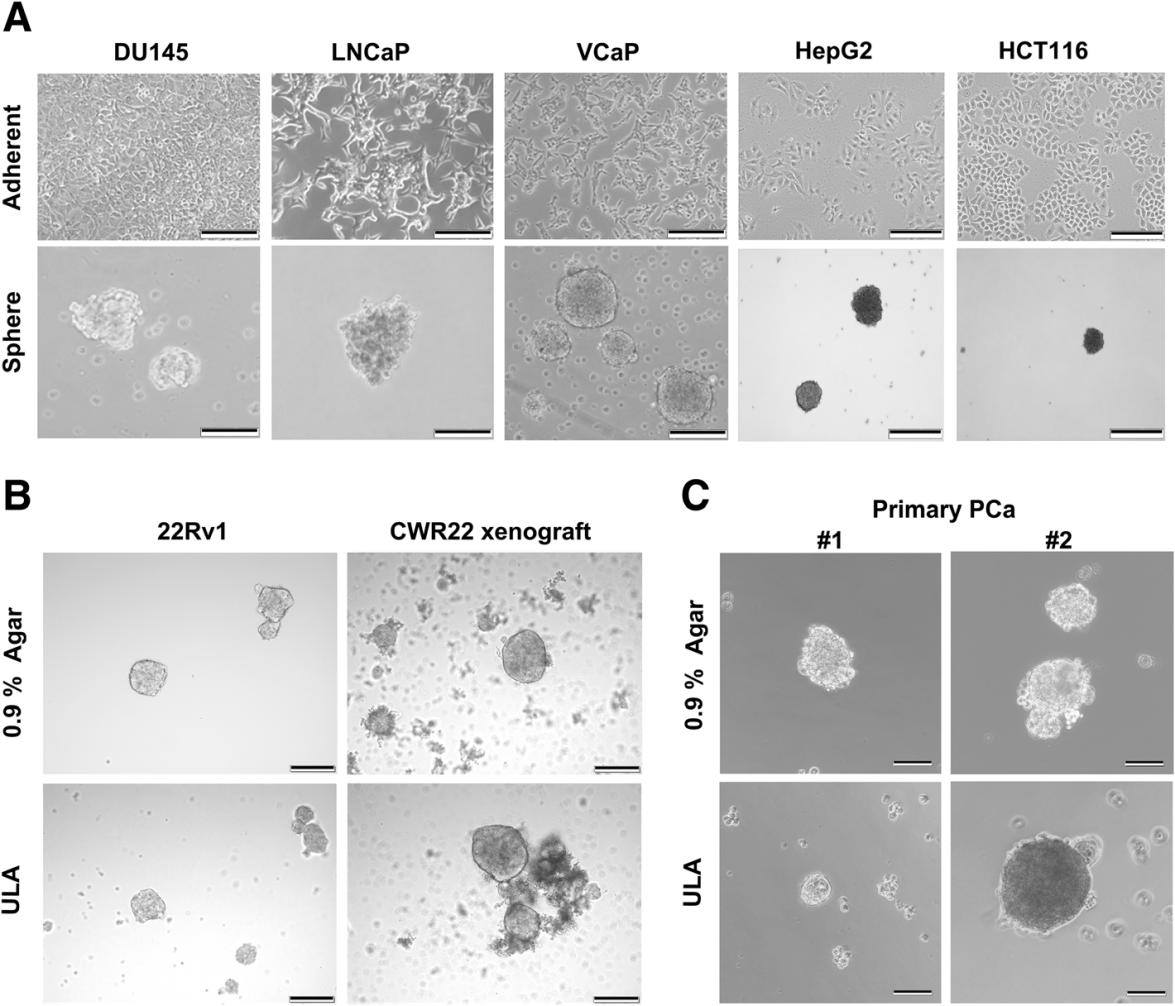
*In vitro* growth of tumor spheres derived from prostatic and non-prostatic cancer cells under adherent two-dimensional (2D) and non-adherent agar-based three-dimensional (3D) culture conditions. **a** Representative images of three prostate cancer cell lines (LNCaP, VCaP, and DU145) and two non-prostatic cancer lines (HCT116 and HepG2) grown under the adherent 2D culture condition and the non-adherent 3D culture condition on agar-coated dishes. Bars: 200 μm. **b** Formation of 3D cultured spheres, derived from the primary human prostate cancer xenograft CWR22 and the 22Rv1 prostate cancer cell line derived from CWR22 xenograft [32], on 0.9% agar plates and commercial ultra-low attachment (ULA) plates. Bars: 200 μm. **c** Images show the tumor spheres formed by primary prostate cancer (PCa) tissues growing on 0.9% agar-coated dishes and ULA dishes for 2 weeks. Bars: 100 μm

**Fig. 3:**
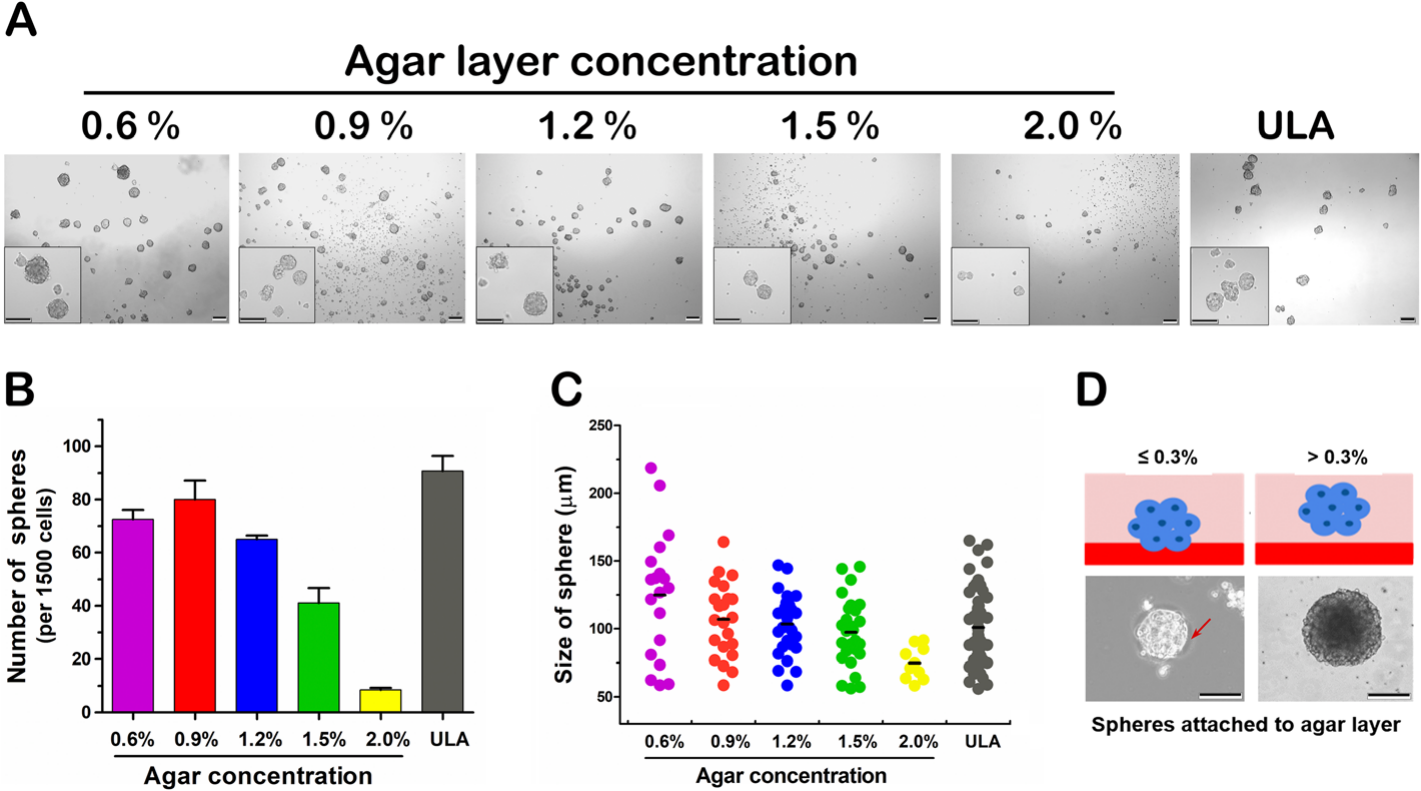
Comparison of growth of prostatospheres formed on dishes coated with different concentrations of agar and commercial ultra-low attachment (ULA) culture dishes. **a** Representative images of spheres derived from single-cell suspensions of DU145 cells (1.5 × 10^3^ cells suspended in 1–2 mL serum-free medium) formed on 0.6–2.0% agar-coated dishes and ULA culture dishes. Inserts show spheres at high magnification. Bars: 200 μm. **b** and **c** Analysis of the number of spheres and their sizes scored on dishes coated with 0.6–2.0% agar and ULA plates. Spheres of at least 50 μm in size were scored under microscope. **d** Upper: schematic diagrams show the growth of sphere into the agar layer in dishes coated with not more than 0.3% agar and its free-suspension growth on dishes coated with at least 0.3% agar. Lower: representative images show the loosely attached spheres on not more than 0.3% agar plates or non-attached spheres formed on at least 0.3% agar-coated plates. Arrow indicates the edges of spheres being grown into the not more than 0.3% soft agar layer. Bars: 100 μm

Non-adherent 3D culture is widely used to isolate SCs from primary cultured normal tissues [9] and the technique is further developed by using various coating agents and used to assess the stemness potential and isolate CSCs from various sources [36]. In this study, we established a novel and low-cost non-adherent culture method for 3D culture of cancer cells or CSCs on the basis of their anchorage-independent growth capacity (Fig. 1). When being 3D-cultured on agar-coated dishes, single-cell suspensions prepared from various cancer cell lines could grow into spheres within 1–3 weeks depending on the cell lines used (Fig. 2a). Moreover, primary cultures of prostate cancer tissues and tumor xenografts could form spheres using the agar-based non-adherent 3D culture method (Fig. 2b and c). We evaluated the coating concentrations of agar for their optimal 3D culture of cancer cell–derived spheres. Analysis showed that there were no significant differences in number and sizes of the spheres, derived from DU145 and VCaP prostate cancer cell lines, formed on dishes coated with agar at concentrations of 0.6–1.2% (Fig. 3a–c). The spheres formed on the agar surface were floating freely upon gentle rocking of dishes as observed under microscope. However, a few growing spheres were observed to be loosely attached to the agar surface or grown into the agar layer upon culture in 0.3% agar-coated dishes but not observed in dishes coated with agar at higher concentrations (Fig. 3d) and this was likely due to the semisolid state or incomplete solidification of agar at low concentration. On the other hand, no spheres were observed being attached to agar surface or grown into agar layer at concentrations of at least 0.6%, suggesting that higher agar concentrations or increased hardness was linked to lower cell adhesiveness or inhibition of attachment. It was also noted that spheres with smaller sizes and number were formed upon culture in dishes coated with a higher percentage (>1.2%) of agar (Fig. 3b and c), suggesting that higher concentrations of agar or increased hardness of agar gel would affect sphere growth. Based on this, 3D culture experiments hereafter were performed in 0.9% agar-coated dishes. We also compared the sphere formation capacity of prostate cancer cells being grown in agar-coated dishes with that in commercially available ultra-low attachment culture dishes. Results showed that DU145 and VCaP cells formed spheres with smooth contour and with equal capacity in numbers and sizes in both agar-coated dishes and commercial ultra-low attachment culture dishes (Fig. 3a–c).

### Prostatospheres formed by agar-based 3D culture exhibit traits of stemness

**Fig. 4:**
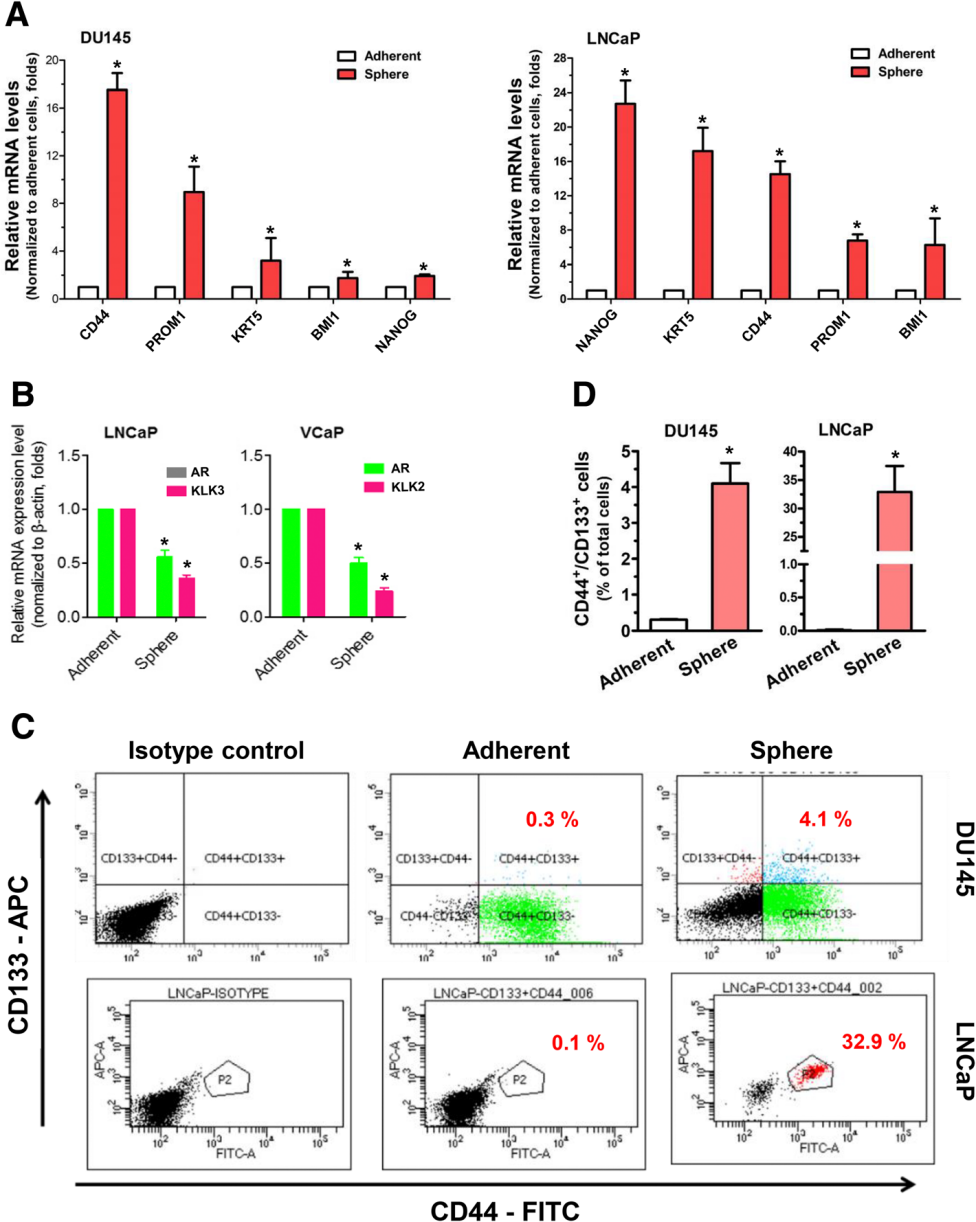
Characterization of stemness phenotype of cancer cell–derived spheres formed on agar-coated dishes. **a** and **b** Reverse transcription quantitative polymerase chain reaction analysis of prostate cancer stem-like cell (PCSC)-associated markers expressed in non-adherent three-dimensional cultured spheres and their corresponding two-dimensional (2D) cultured adherent cells. Results showed that the LNCaP/DU145-derived prostatospheres expressed significantly higher levels of PCSC-associated markers but lower levels of prostate-specific differentiation markers (*AR*, *KLK3/*PSA, and *KLK2*) as compared with their corresponding 2D culture adherent cells. **c** and **d** Fluorescence-activated cell sorting analysis of prostatospheres for CD133 and CD44 expressions. **c** Polychromatic plots and **d** proportion of cell population graphs of CD133^+^/CD44^+^ cells. Results showed that there was a significant increase of CD133^+^/CD44^+^ cell populations in both LNCaP and DU145 prostatospheres as compared with their corresponding 2D culture adherent cells. *P <0.05, compared with corresponding adherent cells.

**Fig. 5:**
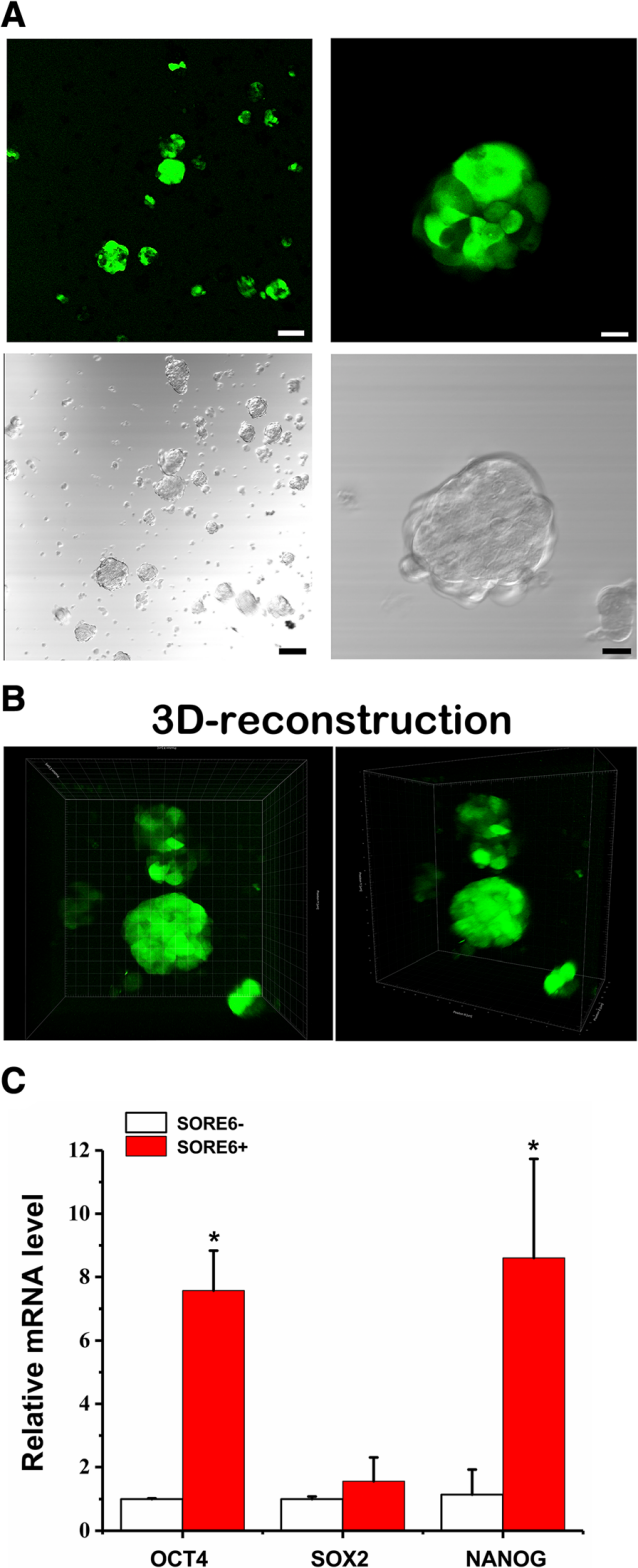
Detection and visualization of prostate cancer stem-like cells (PCSCs) present in three-dimensional (3D) cultured prostatospheres by Sox2/Oct4 response element six tandem repeats (SORE6) reporter system. **a** Fluorescent (upper) and phase contrast (lower) images of DU145-SORE6 cell–derived prostatospheres shown at low (left; bars: 100 μm) and high (right; bars: 20 μm) magnification. **b** z-Stack visualization of individual DU145-SORE6–derived prostatospheres (animation shown in Additional file 1). Results showed that the DU145-SORE6–derived prostatospheres contained SORE6^+^ cells, suggesting that the 3D cultured prostatospheres were enriched of PCSCs. **c** Reverse transcription quantitative polymerase chain reaction analysis of expression levels of *OCT4*, *SOX2*, and *NANOG* in flow cytometry–sorted DU145-SORE6 cells. *P <0.05 SORE6^+^ versus SORE6^−^ cells


The CSCs share a number of characteristics with the normal SCs, such as self-renewal and differentiation capacities [1]. It is known that cellular aggregates or spheres, formed under the non-adherent 3D cultures of single-cell suspensions prepared from different cell or tissue sources, are enriched of stem or progenitor cells [16] and this is likely due to their enhanced anchorage-independent growth capacity and anoikis resistance. Thus, single cell–based and non-adherent 3D culture or sphere formation assay has been widely employed to isolate CSCs, including PCSCs, from various *in vitro* and *in vivo* sources [35, 37, 38]. To validate whether the tumor spheres formed upon agar-based non-adherent 3D culture would contain CSCs, we surveyed the expression profiles of CSC-associated markers by reverse transcription quantitative polymerase chain reaction (RT-qPCR) analysis. Results showed that the agar-based 3D cultured prostatospheres, derived from three prostate cancer cell lines [androgen receptor (AR)-positive: LNCaP and VCaP; AR-negative: DU145], expressed significantly higher levels of PCSC markers (including the commonly expressed CD44, CD133, and *NANOG*) as compared with their counterpart cells grown under the conventional adherent 2D culture conditions (Fig. 4a). On the other hand, RT-qPCR analysis also revealed that differentiation markers, *AR* and its two responsive genes (PSA/*KLK3* and *KLK2*), showed significant downregulation in LNCaP/VCaP-derived prostatospheres as compared with their counterpart 2D cultured adherent cells (Fig. 4b), suggesting that the prostatospheres contained less differentiated prostatic cells. To further validate the enrichment of PCSCs in prostatospheres formed under agar-based 3D culture conditions, we performed the FACS using two PCSC-specific cell surface markers: CD44 and CD133. The results showed that there was a significant increase of CD44^+^/CD133^+^ cell populations in both LNCaP and DU145 prostatospheres (Fig. 4c and d). Together, these results showed that the prostatospheres formed under agar-based SF 3D culture conditions contained enriched numbers of PCSCs. We also used a reporter system SORE6-GFP, which contains multiple tandem repeats of a composite OCT4/SOX2 response element (SORE6) in *NANOG* promoter to drive the expression of reporter GFP [39], to detect and visualize the putative PCSCs in 3D cultured prostatospheres. Our results validated that most of the DU145-derived prostatospheres contained SORE6-reporter responsive cells (SORE6^+^) (Fig. 5a). Confocal microscopic 3D reconstruction and RT-qPCR analyses of the spheres validated that almost all of the cells within spheres were SORE6^+^ cells (Fig. 5b and Additional file 1) and expressed higher levels of *OCT4*, *NANOG*, and *SOX2* (Fig. 5c), further suggesting that the prostatospheres formed in agar-based non-adherent 3D culture were enriched of PCSCs.

Additional file 1:Animation of the 3D reconstruction of DU145-SORE6-GFP spheres. Animation of spinning disc of z-stack reconstruction of 50 confocal optical images of DU145-SORE6-GFP spheres formed by agar-based non-adherent 3D culture method. Abbreviations: *3D* three-dimensional, *SORE6* Sox2/Oct4 response element six tandem repeats. (AVI 8222 kb)


### Repeated use of agar-coated dishes in continuous non-adherent 3D culture

**Fig. 6:**
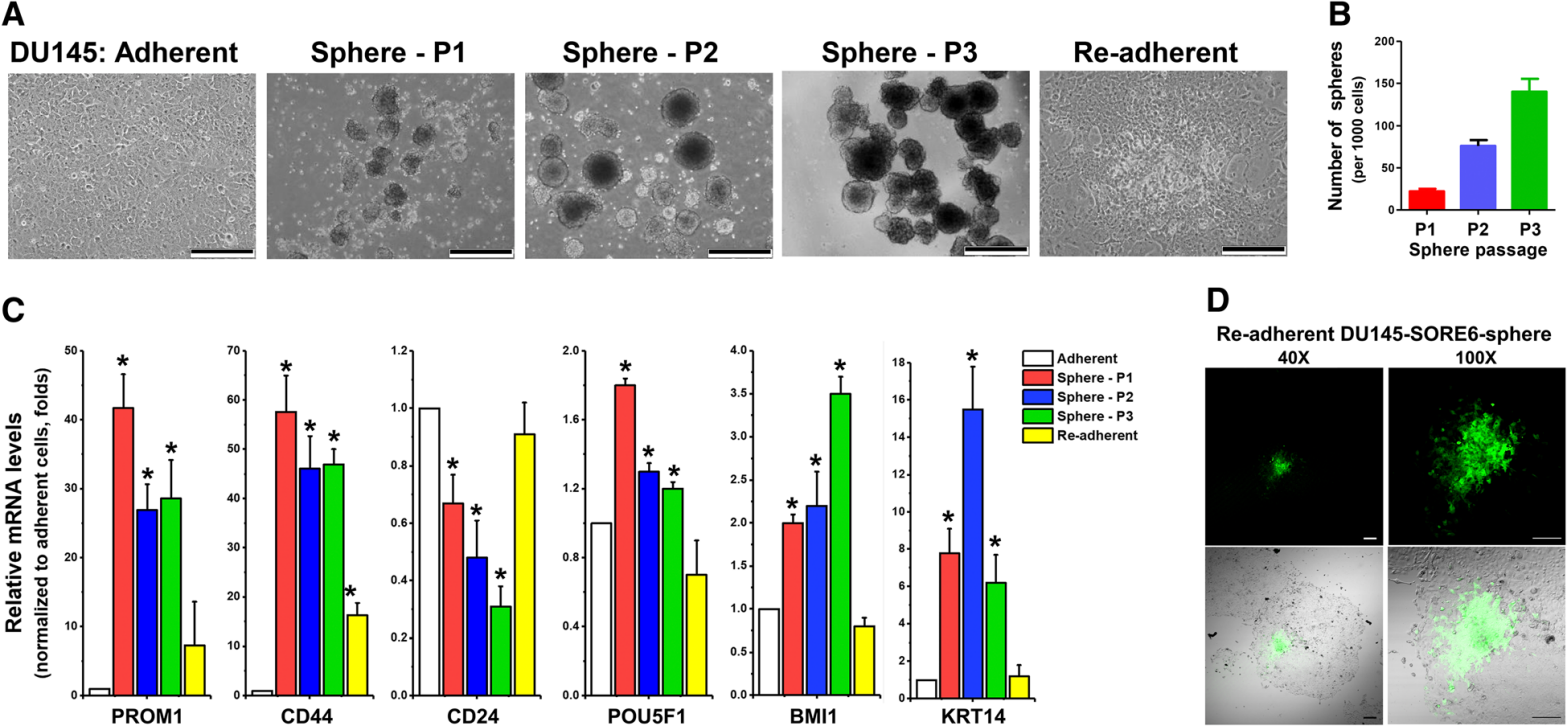
Analysis of sphere formation capacity and expression of prostate cancer stem-like cell (PCSC)-associated biomarkers in three-dimensional culture prostatospheres in serial passages on agar-coated dishes. **a** Representative images of DU145 cells grown under the adherent two-dimensional (2D) culture condition, their corresponding prostatospheres formed upon serial passages P1–P3 on the same agar-coated dishes and 2D culture of re-adherent cells from the last P3-passage prostatospheres. Bars: 200 μm. **b** Sphere formation assay of DU145-derived prostatospheres in serial passages P1–P3. Single-cell suspensions (500 cells) of P1–P3 prostatospheres were seeded in Matrigel-coated 48-well plates and grown for 1–3 weeks followed by enumeration of spheres. Results showed that there was a significant increase of the number of spheres scored in late passages as compared with early passage. **c** Reverse transcription quantitative polymerase chain reaction analysis of PCSC-associated markers expressed in serial passaged DU145 spheres and their corresponding adherent 2D cultured cells. Results showed that the DU145-derived prostatospheres in serial passages expressed significantly higher levels of PCSC-associated markers but lower levels of CD24 as compared with 2D culture adherent cells and re-adherent cells from the last-passage spheres. *P <0.05, compared with corresponding adherent cells. **d** Fluorescent (upper) and fluorescent overlay-phase contrast (lower) images of DU145-SORE6 spheres regrown under adherent 2D culture conditions for 48 h. Bars: 200 μm

The commercially available ultra-low attachment culture dishes are usually coated with a thin layer of non-adhesive hydrogel material of a non-disclosed nature in order to prevent cell attachment in non-attachment or suspension 3D cultures. Repeated use of such culture dishes for continuous cell maintenance is not recommended by manufacturers as cleansing of dishes will remove or damage the coating that will result in cell attachment in subsequent cultures. Thus, single use of such dishes as advised is the major cost in non-attachment 3D cultures. Here, we sought to examine the possible repeated use of agar-coated dishes or plates for continuous 3D culture and maintenance of cells in a suspended state in order to further reduce the cost and labor of preparation of agar-coated dishes. The DU145-derived prostatospheres were allowed to grow in agar-coated dishes for 1–3 weeks to reach sizes of at least 50 μm, harvested, dissociated by TrypLE treatment into single-cell suspensions, and re-plated onto the same agar-coated dishes, which had been briefly pre-rinsed with PBS to remove any residual cells, at low cell density. Under this condition, the spheres grown in re-used agar-coated dishes showed no difference in their morphology as compared with those spheres grown in freshly prepared dishes (Fig. 6a). The re-suspended spheres were subcultured in the same agar-coated dishes up to six serial passages, and no spheres were observed being attached to the agar surface or grown into the agar layer. For comparison, we also performed the serial passages of spheres on the same ultra-low attachment culture dishes. Our results showed that when the ultra-low attachment culture dishes were reused, some spheres were observed being attached to the bottom surface of the plates in the second and third passages (results not shown).

### Continuous passages of prostatospheres can enhance the sphere formation capacity of prostate cancer cells

We next evaluated the sphere formation capacity of DU145-derived prostatospheres in their serial passages in agar-based non-adherent 3D culture. The sphere formation capacity of the DU145 prostatospheres at the first passage (P1) was shown to be 4.4%. In the subsequent serial passages of spheres, the sphere formation capacity was significantly increased to 15.2% in P2 spheres and 28% in P3 spheres (Fig. 6b), suggesting that the PCSC population could be further enriched by continued 3D culture of the spheres. Expression analysis by RT-qPCR also showed that the expression levels of PCSC-associated markers (including *PROM1/*CD133, *CD44*, *KRT14*, *BMI1*, and *POU5F1*/OCT4) showed significant upregulation, whereas another marker *CD24* exhibited passage-dependent downregulation, in P1–P3 spheres (Fig. 6c). CD44^+^/CD24^−^ phenotype is characterized to be an expression marker for PCSCs [40]. Comparison of the upregulated PCSC markers showed no significant difference between the P1 spheres and spheres at higher passages, suggesting that the PCSCs present in the spheres or their self-renewal capacity were maintained in stable status during the serial passages. When the spheres formed in late passages were subcultured in 2D monolayer adherent culture conditions, the re-adherent cells showed decreased expressions of PCSC markers at levels comparable to those of the original 2D cultured cells, suggesting that the PCSCs in the serial passaged spheres possessed the capacity of differentiation into differentiated tumor cells, a hallmark of CSCs. Furthermore, to examine the differentiation potency of PCSC-enriched prostatospheres, the spheres derived from SORE6-GFP cells were re-grown under adherent 2D culture conditions. The results showed that upon attachment to the culture-ware surface, cells from spheres migrated out and spread on the surface with loss of SORE6-activated GFP signals, indicating that the SORE6^+^ cells (PCSCs) were undergoing differentiation into SORE6^−^ cells upon re-attachment to the surface in adherent 2D culture (Fig. 6d).

### Prostatospheres formed by agar-based 3D culture show enhanced *in vivo* tumorigenicity

**Fig. 7:**
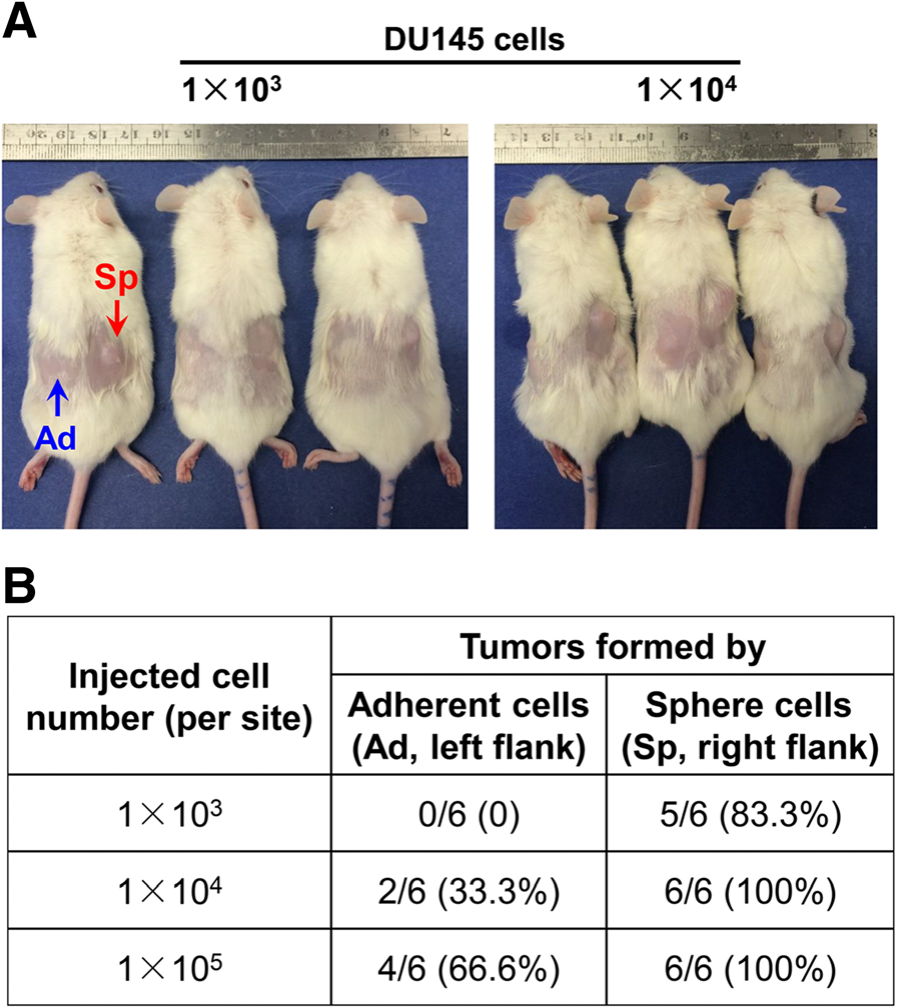
Comparison of tumor formation capacity of DU145 cells derived from three-dimensional (3D) culture spheres and adherent two-dimensional (2D) culture. **a** Photographs of representative severe combined immunodeficiency mice bearing the subcutaneous xenograft tumors formed by the inoculated DU145 cells derived from either the 3D culture spheres (Sp, right flank) or adherent 2D culture (Ad, left flank), with inoculated cell numbers of 1 × 10^3^ or 1 × 10^4^ cells per site. **b** Table summarizes the results of xenograft tumors formed in mice receiving inoculated DU145 cells derived from 3D culture spheres and adherent 2D culture (with inoculated 1 × 10^3^, 1 × 10^4^, and 1 × 10^5^ cells per site; *n* = 6 for each experimental group). Cells derived from 3D culture spheres showed significantly higher tumor formation capacity in mice than cells from adherent 2D culture with either low or high inoculation cell numbers

High tumor formation capacity is a major characteristic of CSCs. We next performed the *in vivo* tumorigenicity assay to evaluate the tumor formation capacity of DU145 cells prepared from prostatospheres formed by agar-based non-adherent 3D culture and adherent 2D culture in intact SCID mice. DU145 cells prepared from 3D cultured spheres and injected at high cell numbers of 1 × 10^4^ or 1 × 10^5^ showed 100% tumorigenicity in host mice as compared with only 33.3% and 66.6% tumorigenicity for cells prepared from 2D adherent culture with the same inoculation cell numbers. With low cell number injection (1 × 10^3^), cells prepared from 3D cultured spheres also showed significantly higher tumorigenicity (100%) as compared with cells prepared from 2D adherent culture, which formed no tumor in host mice (Fig. 7). The results showed that the non-adherent 3D cultured spheres were enriched of PCSCs and showed significantly higher tumor-initiating capacity as compared with cells derived from the 2D adherent culture.

## Discussion

In past decades, different non-adhesive substrata or coating agents have been used in non-adherent suspension 3D cultures or anchorage-independent clonogenic assay, including polymers of 2-hydroxyethyl methacrylate (poly-HEMA) [41–44], synthetic polymer-derived hydrogels (for example, polyethylene glycol) [45], agar [20, 46, 47], and agarose [48, 49]. These non-adhesive coating agents used in non-adherent culture assay have demonstrated their usefulness of growing multicellular tumor spheroids. However, preparation of these non-adherent plates is either complicated or labor-intensive (for example, even coating of poly-HEMA) and also the cost of commercial non-adherent culture-ware is relatively expensive. As shown in this study, agar coating can offer a number of advantages in non-adherent 3D culture for CSC enrichment, including its low cost, simplicity in preparation, and reusability for serial passages, as compared with other non-adhesive coating agents and non-culture CSC isolation methods (Table 2). However, its only disadvantage is that the agar-coated plates or dishes cannot be kept for a long time (over 2 weeks), as drying of agar coating may affect the culture condition.

**Table 2:** Comparison of methods for isolation and enrichment of cancer stem-like cells

Methods	Cost	Time of operation	Advantages	Disadvantages
Agar-based non-adherent 3D culture	Very low	<2 h	Simple procedure; reusable	Freshly prepared required
3D culture in commercial ultra-low attachment dishes	High	None	Ready to use; long-time storage	Not reusable
Poly-HEMA–based non-adherent 3D culture	Low	>8 h	Long-time storage	Complicated preparation procedure, not reusable
Antibody-based FACS and MACS	High	>3 h	High-quality cancer stem-like cells	Markers are cell type–dependent, complicated procedure
Scaffold-supported 3D culture (for example, Matrigel)	High	>2 h	Microenvironment mimicking	Potential differentiation-induced, temperature-sensitive solidification
Side population sorting	Medium	>3 h	Functional relevant	Inconsistent results

Abbreviations: *3D* three-dimensional, *FACS* fluorescence-activated cell sorting, *MACS* magnetic-activated cell sorting, *poly-HEMA* poly-2-hydroxyethyl methacrylate

Intriguingly, we observed in this study that continuous passage of tumor spheres could enhance the sphere formation capacity of prostate cancer cells. However, it remains to be determined whether this change in sphere formation capacity is due to further selection of the subpopulation of CSCs or other causes. In the initial passages of tumor spheres, the anchorage-dependent non-CSCs decreased in their numbers because of anoikis whereas the anoikis-resistant CSCs increased in number correspondingly, thus contributing to increased sphere formation capacity upon continuous passages. So far, little is known about the significance of long-term or continuous culture of CSCs in terms of their phenotypic changes. It has been shown that prolonged 3D culture of mammospheres can induce the epithelial-to-mesenchymal transition in MCF-7 breast cancer cells [50]. It is well known that continuous or prolonged passage of cancer or immortalized cell lines can lead to certain phenotype and genetic changes induced by number of causes, such as chromosomal instability [51], progressive or subclone selection [52, 53], and altered expressions of cell cycle regulators and oncogenes [54]. Prolonged culture of embryonic and pluripotent SCs can induce genetic and epigenetic alterations mediated by different casual mechanisms [55, 56].

Many studies indicate that *in vitro* scaffold-supported 3D cultures, particularly using natural ECMs (for example, collagen and Matrigel), can closely mimic the *in vivo* microenvironment status or recapitulate certain physiological features of tissues or tumors, as the scaffold culture conditions can promote cell differentiation and survival through proper cell–ECM and cell–cell interactions [13]. Interactions with different ECM components can have a different impact on cell growth and behaviors in 3D cultured cells. For example, 3D culture of MCF-7 breast cancer cells in collagen gel can promote the growth of CSCs [12], whereas 3D culture of human mammary epithelial cells or induced pluripotent SCs in Matrigel can induce and enhance their respective differentiation phenotypes [57, 58]. It has also been shown that the relative stiffness of different supporting ECMs can exert different effects on cancer SC growth and their behaviors in 3D cultured hepatocellular carcinoma cells [59]. Indeed, we observed that 3D culture of cancer cells in Matrigel showed a higher sphere formation capacity than agar-based suspension 3D culture of the same cell types (not shown). However, the number of CSCs present in the tumor spheres generated by different 3D culture methods may be different. Using the SORE6 reporter system, we observed that the tumor spheres generated by the agar-based suspension 3D culture contained more CSCs (SORE6^+^ cells) than the spheres generated by Matrigel-based 3D culture, suggesting that the absence of interaction with ECM in non-adherent 3D culture may favor the growth of CSCs or that the multi-cellular tumor spheroids generated by ECM-based 3D cultures may contain more non-CSCs or less CSCs.

## Conclusions

In summary, we developed and optimized a novel non-adherent 3D culture method based on agar-coating for growing multicellular tumor spheres derived from different prostatic and non-prostatic cancer cell lines and also primary prostate cancer tissues. We also demonstrate its usefulness in the isolation and enrichment of proliferating CSCs, and also serial passages of tumor spheres under non-adherent and SF culture conditions can help to maintain their undifferentiation features and further enhance their sphere formation capacity. The present method offers several advantages as compared with other non-adherent coating agents used in non-adherent 3D cultures and other non-culture CSC isolation methods, including its simplicity, reproducibility, low cost without the use of expensive equipment (for example, flow cytometer) and specific antibodies, and the repeated use of the agar-coated plates for serial passages of CSC-enriched spheres.

## Abbreviations

2D: Two-dimensional
3D: Three-dimensional
ALDH1: Aldehyde dehydrogenase isoform 1
AR: Androgen receptor
CSC: Cancer stem-like cell
DMEM/F12: Dulbecco’s modified Eagle’s medium/Nutrient Mixture F-12
ECM: Extracellular matrix
FACS: Fluorescence-activated cell sorting
FBS: Fetal bovine serum
GFR: Growth factor reduced
MACS: Magnetic-activated cell sorting
P: Passage
PBS: Phosphate-buffered saline
PCR: Polymerase chain reaction
PCSC: Prostate cancer stem-like cell
Poly-HEMA: Poly(2-hydroxyethyl methacrylate)
PSA: Prostate-specific antigen
RT-qPCR: Reverse transcription quantitative polymerase chain reaction
SC: Stem cell
SCID: Severe combined immunodeficiency
SF: Serum-free
SORE6: Sox2/Oct4 response element six tandem repeats
SP: Side population
